# Calpains of *Leishmania braziliensis*: genome analysis, differential expression, and functional analysis

**DOI:** 10.1590/0074-02760190147

**Published:** 2019-09-23

**Authors:** Vítor Ennes-Vidal, Bianca da Silva Vitório, Rubem Figueiredo Sadok Menna-Barreto, André Nóbrega Pitaluga, Silvia Amaral Gonçalves-da-Silva, Marta Helena Branquinha, André Luis Souza Santos, Claudia Masini d’Avila-Levy

**Affiliations:** 1 Fundação Oswaldo Cruz-Fiocruz, Instituto Oswaldo Cruz, Laboratório de Estudos Integrados em Protozoologia, Rio de Janeiro, RJ, Brasil; 2 Fundação Oswaldo Cruz-Fiocruz, Instituto Oswaldo Cruz, Laboratório de Biologia Celular, Rio de Janeiro, RJ, Brasil; 3 Fundação Oswaldo Cruz-Fiocruz, Instituto Oswaldo Cruz, Laboratório de Biologia Molecular de Parasitas e Vetores, Rio de Janeiro, RJ, Brasil; 4 Universidade do Estado do Rio de Janeiro, Laboratório de Imunofarmacologia Parasitária, Rio de Janeiro, RJ, Brasil; 5 Universidade Federal do Rio de Janeiro, Laboratório de Estudos Avançados de Microrganismos Emergentes e Resistentes, Rio de Janeiro, RJ, Brasil

**Keywords:** cysteine peptidases, calcium-dependent peptidase, leishmaniasis, trypanosomatid

## Abstract

**BACKGROUND:**

Calpains are proteins belonging to the multi-gene family of calcium-dependent cysteine peptidases that undergo tight on/off regulation, and uncontrolled proteolysis of calpains is associated with severe human pathologies. Calpain orthologues are expanded and diversified in the trypanosomatids genome.

**OBJECTIVES:**

Here, we characterised calpains in *Leishmania braziliensis*, the main causative agent of cutaneous leishmaniasis in Brazil.

**METHODS/FINDINGS:**

In total, 34 predicted calpain-like genes were identified. After domain structure evaluation, reverse transcription-quantitative polymerase chain reaction (RT-qPCR) during *in vitro* metacyclogenesis revealed (i) five genes with enhanced expression in the procyclic stage, (ii) one augmented gene in the metacyclic stage, and (iii) one procyclic-exclusive transcript. Western blot analysis revealed that an antibody against a consensus-conserved peptide reacted with multiple calpain-like proteins, which is consistent with the multi-gene family characteristic. Flow cytometry and immunocytochemistry analyses revealed the presence of calpain-like molecules mainly in the cytoplasm, to a lesser extent in the plasma membrane, and negligible levels in the nucleus, which are all consistent with calpain localisation. Eventually, the calpain inhibitor MDL28170 was used for functional studies revealing (i) a leishmaniostatic effect, (ii) a reduction in the association index in mouse macrophages, (iii) ultra-structural alterations conceivable with autophagy, and (iv) an enhanced expression of the virulence factor GP63.

**CONCLUSION:**

This report adds novel insights into the domain structure, expression, and localisation of *L. braziliensis* calpain-like molecules.

The various species of *Leishmania* include parasites of considerable medical and economic importance. Each year, there are 1.5-2 million new estimated cases of leishmaniasis with around 70,000 deaths, and 350 million people are at risk of infection and disease. This disease is characterised by a spectrum of clinical manifestations ranging from cutaneous ulcers to deadly visceral lesions. The present therapy for leishmaniasis is limited to few drugs that are associated with disadvantages such as unacceptable toxicity, difficulties during administration, and treatment failure.^1^ ^,^ ^2^

*Leishmania* parasites are transmitted to human and animals by the bite of a phlebotominae insect. In the mammalian host, the parasite has an obligate intracellular form, namely amastigotes, whereas, in the invertebrate host, in a process known as metacyclogensis, the promastigotes differentiate from a replicating procyclic to a non-replicating infective metacyclic stage.^2^ *Leishmania* peptidases, a class of hydrolytic enzymes responsible for breaking peptide bonds, contribute to essential steps of the parasite life cycle, such as the simple digestion of proteins for nutrition, proliferation and growth, differentiation, signalling, death pathways, and mediating and sustaining the infectious disease process.^3^

Calpains (EC 3.4.22.17, Clan CA, family C02) belong to a family of intracellular Ca^2+^-dependent cysteine peptidases, initially described and characterised in humans. These peptidases are more likely to act in limited proteolysis to slightly modify their substrates and modulate several cellular processes than in full protein digestion; hence, they are designated as intracellular ‘modulator peptidases’, participating in cytoskeletal rearrangement, signal transduction pathways, and apoptosis. Calpain deregulation is associated to several pathologies such as muscular dystrophies, diabetes and tumorigenesis in humans, embryonic lethality in mouse and incomplete sex determination in nematodes. These aspects led to the development of a broad range of selective calpain inhibitors, which can be assayed under a re-purpose strategy in trypanosomatids.^5^

Calpain homologues are identified based on the primary sequence characteristics of the cysteine peptidase core (CysPc), which have been increasingly found in other organisms including insects, nematodes, protozoa, plants, fungi and even in some bacteria, thus constituting a super-family with versatile functions.^4^ ^,^ ^6^ In trypanosomatids, this gene family is expanded and a high diversity is observed in the domain arrangements, ranging from proteins with only one small domain, known as small kinetoplastid calpain-related proteins (SKCRPs), to large proteins comprising four domains, including the classical CysPC.^5^ ^,^ ^7^ ^,^ ^8^ In *Trypanosoma brucei*, the role of this gene family in cytoskeleton rearrangement, with impact on parasite growth, morphology and flagellum assembly, has been proved.^9^ In *Leishmania* spp., an increased expression of this gene family members was associated to drug-resistance, post-kala-azar dermal leishmaniasis and metacyclogenesis.^5^ ^,^ ^10^ ^,^ ^11^ ^,^ ^12^ The calpain inhibitor, MDL28170 (inhibitor III, Z-Val-Phe-CHO), induces apoptotic marker expression in *Leishmania amazonensis* ^8^ and impairs promastigote proliferation and amastigote intracellular development.^5^ ^,^ ^8^ ^,^ ^13^

Although *Leishmania* species form a monophyletic clade, differences in these species can account for distinct disease outcomes and vector specificity.^14^ *L*. *braziliensis* is associated with mucosal and disseminated leishmaniasis to a greater extent than other New World *Leishmania*, and is the most widely distributed causative agent of cutaneous leishmaniasis in Brazil.^1^ ^,^ ^2^ Therefore, owing to the severity and public health importance of *L. braziliensis*, the Drugs for Neglected Diseases initiative (DND*i*) decided to focus on the development of novel treatment options for cutaneous leishmaniasis, predominantly caused by this species.^15^ Thus, the study of calpain molecules and the *in vitro* evaluation of an alternative treatment, not assayed yet, against this *Leishmania* species are extremely relevant. Moreover, as the up-regulation of several members of the calpain family leads to a diverse range of biological processes and human diseases, this peptidase family proves to be an important therapeutic target, and it has been immensely explored for the development of a means of identifying selective calpain inhibitors.^5^ ^,^ ^8^ Further studies about trypanosomatid calpains may employ calpain inhibitors developed to treat human pathologies, and selectivity may not be essential for anti-protozoan drugs due to the inherent biological selectivity in the function and location of the protozoan peptidases.^3^ In addition, the inhibitor concentration necessary to chemically knock-out a parasitic enzyme is presumably much lower than that predicted for the homologous host enzymes.^3^ ^,^ ^5^ Collectively, the knowledge of structural and functional relationships and substrate specificity of these proteins in trypanosomatids should make them ideal candidates for computation-assisted drug design for specific inhibitors. In the present study, we screened *L. braziliensis* whole genome to identify and classify the calpain genes and their domain arrangements; thereafter, we evaluated the gene expression pattern between procyclic and metacyclic promastigotes during *in vitro* metacyclogenesis. The protein profile and cellular localisation was assessed by means of a polyclonal antibody raised against a consensus-conserved region of the CysPC domain. Eventually, MDL28170 was employed to address its effect on the parasite growth, ultra-structure, and interaction with mouse peritoneal macrophages, as well as its effects on the abundance of calpain-like proteins and two well-known leishmania virulence factors, cpb and GP63.^3^

## MATERIALS AND METHODS

*Calpain search in L. braziliensis genome, conserved domain analysis, gene selection and primer design* - Protein sequences of *L. braziliensis* MHOM/BR/75/M2904 strain annotated as calpains were retrieved from the Tritryp Database. These proteins were locally analysed by Simple Modular Architecture Research Tool (SMART) for the presence of calpain domains in InterPro and Pfam databases. In addition, an HMM model was created with a wide range of annotated calpains and an HMM search was done in *L. braziliensis* genome (GeneBank ID 718). Sequences containing less than 100 amino acid residues and domains with e-value higher than 10^-3^ were removed from the analysis. Gene-specific primers of sequences harbouring the calpain proteolytic core (CysPc) were designed using Primer3Plus to amplify a 90-120 bp fragment in quantitative polymerase chain reaction (qPCR) analysis [Supplementary data (Table)]. The predicted molecular mass of calpain sequences was calculated using Bioinformatics.org (http://www.bioinformatics.org/sms/prot_mw.htm).

*Parasite cultivation and procyclic and metacyclic isolation* - *L. braziliensis* promastigotes (strain Thor, MCAN/BR/1998/619) were routinely inoculated in hamster and re-isolated from their lesions and were subsequently maintained (up to 4 passages - recently isolated strain - RI) in Schneider’s medium at 26ºC. Culture adapted strains (CAS) had at least 30 sub-cultures *in vitro*. To separate the procyclics from metacyclic promastigotes, the stationary phase culture was subjected to differential centrifugation in Ficoll® 20%.^16^ Next, each population was analysed via flow cytometry through the forward scatter. Experiments were carried out in accordance with the protocols approved by the Institutional Animal Care and Use Committee at Instituto de Biologia Roberto Alcântara Gomes of the Universidade Estadual do Rio de Janeiro (UERJ) (CEUA/051/2017).

*Gene expression comparison between procyclic and metacyclic L. braziliensis* - Total RNA from the procyclic and metacyclic stages was extracted using TRIzol reagent, according to the manufacturer’s instructions. RNA samples were treated with DNAse I to remove any contaminating DNA and were then analysed for purity and were quantified in a spectrophotometer. The cDNA synthesis was performed with SuperScriptIII kit (Applied Biosystems) using oligo-dT primers. The specificity of each designed primer was confirmed by sequencing the amplified products by Sanger sequencing in an ABI 3730 Sequencing Platform. The DNA sequences were evaluated against NCBI nr database using BLASTn. For qPCR, cDNA was diluted 10 times and was used in 20 µL reaction including Go-Taq PCR Master Mix and primers in ABI Prism 7500 FAST (Applied Biosystem). The relative gene expression was determined using comparative CT values. ΔCT of the target gene was obtained as a difference in the CT value from the endogenous control actin. The constitutive 8S gene was additionally analysed to improve data confidence. The ΔΔCT value of each gene was calculated pair-to-pair between the procyclic and metacyclic stages, and the one with higher reduction in the expression level was regarded as the reference. The relative expression was then reported as 2^-ΔΔCT^.^17^

*Anti-calpain polyclonal antibody production* - *L. braziliensis* calpains alignment through MUSCLE v3.8.31 allowed the identification of a consensus conserved unique polypeptide, LEKAYAKLHGSY, among the 34 calpain sequences. This peptide was synthesised and used to immunise rabbits to obtain a polyclonal serum by Rhea Biotech Enterprise (Campinas, São Paulo, Brazil). The antibody was purified by affinity chromatography. The antibody ‘anti-tritryp-calpain’ presented a final concentration of 0.1 mg/mL.

*Flow cytometry analysis* - The total promastigotes (1.0 × 10^6^ cells) of either RI or CASlog-phase cultures were processed and analysed for flow cytometry, as previously described.^18^ Briefly fixed parasites in paraformaldehyde (0.4%), permeabilised or not with Triton X-100 (0.01%), were incubated at room temperature for 1 h with anti-tritryp-calpain polyclonal antibody (1:250 dilution). Alternatively, the RI cultures were incubated with MDL28170 at 3.3, 6.6, and 13.2 μM for 48 h and were then fixed and processed as described above, and were additionally labelled with anti-cpb (kindly provided by Dr Mary Wilson, Department of Internal Medicine, Biochemistry, Microbiology and Epidemiology, Program in Molecular Biology, University of Iowa, USA) (1:500 dilution) or anti-GP63 (kindly provided by Dr Peter Overath, Max-Planck-Institutfür Biologie, Abteilung Membranbiochemie, Germany) (1:1000 dilution). Under all conditions, the cells maintained their morphological integrity, as verified by the optical microscopic observation. After incubation with the primary antibodies, the cells were treated with Alexa 488-labelled goat anti-rabbit IgG (1:750 dilution) for 1 h at room temperature, and data acquisition and analysis were performed on a flow cytometer equipped with a 15 mW argon laser emitting 488 nm wavelength (FACS Calibur, BD Bioscience, USA). The omission of the primary antibody was used as a control. Each experimental population was then mapped by using a two-parameter histogram of forward-angle light scatter versus side scatter. The mapped population (n = 10,000) was then analysed for log green fluorescence by using a single parameter histogram, and the mean fluorescence intensity (MFI) of each experimental system was divided by MFI from the auto-fluorescence controls to obtain the variation index.

*Identification of calpains by Western blotting* - The total promastigotes (1.0 × 10^8^ cells) of either RI or CAS log-phase cultures were processed for immunoblotting analysis, as previously described.^18^ The nitrocellulose membranes comprising the transferred proteins were blocked in 10% low-fat dried milk dissolved in PBS containing 2% Tween 20 (TBS/Tween) overnight at 4ºC, and were then washed with the blocking solution and subsequently incubated for 2 h with anti-tritryp-calpain (1:500 dilution). To analyse the antibody specificity, the anti-tritryp-calpain was incubated for 2 h in an ELISA plate coated with LEKAYAKLHGSY or an unrelated polypeptide (FGFVEEGAEERKAVAELKK). The supernatant of these reactions was collected and incubated in an additional blotting membrane with *L. braziliensis* proteins. The secondary peroxidase-conjugated goat anti-rabbit immunoglobulin G (1:1500 dilution) was used followed by chemiluminescence immunodetection. An anti-β-actin polyclonal antibody produced in rabbit (Rhea Biotech) (1:5000 dilution) was used as a loading control. The relative molecular masses of the reactive polypeptides were calculated by comparing with the mobility of sodium dodecyl sulfate polyacrylamide gel electrophoresis (SDS-PAGE) standards, and the densitometric analysis was performed using the ImageJ program.

*Multiplication inhibition assay* - The effects of MDL28170 (Calbiochem, San Diego, CA, USA) on *L. braziliensis* promastigotes were assessed as previously described with slight modifications.^18^ Briefly, total promastigotes of either RI or CAS log-phase cultures were enumerated using a Neubauer chamber and were re-suspended in a fresh medium to a final concentration of 1.0 × 10^6^ viable promastigotes/mL. The inhibitor compound was added to the culture at final concentrations of 1.25, 2.5, 5, 10, and 20 µM (starting from a 500 mM solution in dimethyl sulphoxide (DMSO) that was serially diluted in the culture medium). Dilutions of DMSO corresponding to those used to prepare the drug solutions were assessed in parallel. After incubation (24-96 h) at 26ºC, the number of viable motile promastigotes was quantified in a Neubauer chamber. Alternatively, protozoa grown for 48 h in the absence or presence of the calpain inhibitor were washed thrice in PBS prior to re-suspension in a drug-free fresh medium and were allowed to grow for another 96 h to evaluate the leishmanicidal or leishmanistatic effect. The live motile promastigote count was evaluated under optical microscopy at 24 h intervals. The 50% inhibitory concentration (IC_50_), that is, the drug concentration that caused a 50% reduction in survival in comparison to that in identical cultures without the compound, was calculated daily.

*Interaction between murine macrophages and L. braziliensis* - Peritoneal macrophages were extracted from BALB/c mice and adhered in coverslips (3 × 10^5^ cells) with RPMI 1640 medium in a 4% CO_2_ atmosphere at 37ºC for 24 h, and were then washed thrice with PBS. The RI parasites from the stationary growth phase were harvested via centrifugation, washed twice with PBS, and then washed once with RPMI 1640 medium. Thereafter, the parasites were pre-incubated for 1 h in Schneider’s insect medium in the absence or presence of MDL28170 at final concentrations of 3.3, 6.6 and 13.2 μM. Parasite viability remained unaffected in this condition, as presented by trypan blue dye exclusion and cellular motility. DMSO (at the highest concentration used as a drug diluent) was assessed in parallel. Parasites fixed in 4% paraformaldehyde were included as an additional control and were allowed to interact with macrophages for 1 h (10 parasites per host cell). Thereafter, the unbound parasites were removed by washing with PBS, and the coverslips were fixed and stained with Panotico kit (Laborclin, Paraná, Brazil). Cells were observed by light microscopy, and the percentage of infected macrophages was determined by randomly counting at least 200 cells in each biological sample. The association index was obtained by multiplying the percentage of infected macrophages by the number of amastigotes per infected macrophage. Experiments were carried out in accordance with the protocols approved by the Institutional Animal Care and Use Committee at FIOCRUZ (CEUA LW 16/13).

*Transmission electron microscopy analyses* - Briefly, *L. braziliensis* RI promastigotes (1.0 × 10^6^ cells/mL) were treated or untreated at final concentration of 3.3 µM MDL28170 in Schneider’s insect medium at 26ºC. Thereafter, the parasites were fixed with 2.5% glutaraldehyde in 0.1 M Na-cacodylate buffer (pH 7.2) at room temperature for 40 min and were post-fixed with a solution of 1% OsO_4_, 0.8% potassium ferricyanide and 2.5 mM CaCl_2_ in the same buffer for 20 min at room temperature.^18^ Parasites were dehydrated in an ascending acetone series and were embedded in PolyBed 812 resin. Ultra-thin sections were stained with uranyl acetate and lead citrate and were examined in Jeol JEM1011 transmission electron microscope at Plataforma de Microscopia Eletrônica, IOC, FIOCRUZ. Alternatively, the untreated parasites were subjected to pre-embedding protocol, in which the parasites were fixed, permeabilised (Triton X-100 0.1%) and incubated with a 1:20 dilution of polyclonal rabbit anti-tritryp-calpain, followed by labelling with a 1:40 dilution of the secondary anti-rabbit-gold (10 nm) antibody.

*Statistical analysis* - All experiments were repeated at least thrice and were performed in triplicate and the results represent the media and standard deviation. When appropriate, the representative images of the experiments are depicted. The data were analysed statistically by Student’s *t* test using GraphPad Prism Version 5.00 software. *P* values of 0.05 or less were considered as statistically significant.

## RESULTS AND DISCUSSION

*A large diversity of domain arrangement revealed in L. braziliensis calpain family -* To date, the diversity of the calpain gene family has been mainly scrutinised in multi-cellular complex eukaryotes, such as animals and plants. Nevertheless, the calpain-related genes have also been reported in microorganisms, including trypanosomatids. Interestingly, a great difference in the gene number and domain diversity is observed among microorganisms and mammals, and the divergent evolutionary histories may account for this diversity.^6^ Here, we evaluated the presence of calpain-related genes in *L. braziliensis* genome. Using two distinct search approaches, 34 calpain-related genes distributed in 13 different chromosomes were retrieved, revealing a wide range of domain arrangements (Table). Combinations between the proteolytic core domain (CysPc) and several other domains, such as small kinetoplastid calpain-related proteins (SKCRPs), microtubule interacting domain (MIT), regular structure consisting of similar repeats (RNI-like), and repetitive-rich regions, revealed the wide variation in calpain-like member domain architecture, as previously observed by Zhao et al.^6^ in unicellular eukaryotes.

**TABLE:** List of calpain sequences retrieved from *Leishmania braziliensis* genome

Gene ID	Chromosome	Domain architecture	Consensus peptide sequence (LEKAYAKLHGSY)	Predicted Molecular mass (kDa)
LbrM.04.0490	4	SKCRP, CysPc	LEKAFAKMHGSY	94
LbrM.14.0820	14	SKCRP	----------	13
LbrM.14.0830	14	SKCRP	----------	13
LbrM.17.1220	17	KISC, 3xARM, CBSW	----------	158
LbrM.18.1160	18	CysPc	*Low similarity*	84
LbrM.20.0290	20	CysPc	*Low similarity*	174
LbrM.20.5340	20	SKCRP	----------	38
LbrM.20.5380	20	SKCRP, CysPc	LEKAYAKLHGSY	91
LbrM.20.5390	20	Fragmented CysPc	*Low similarity*	23
LbrM.20.5400	20	SKCRP, CysPc	LEKAYAKIFGGY	78
LbrM.20.5410	20	SKCRP, CysPc	*Low similarity*	83
LbrM.20.5430	20	CysPc	LEKAYAKVRGGY	60
LbrM.20.5440	20	SKCRP	----------	17
LbrM.20.5450	20	SKCRP	----------	17
LbrM.20.5500	20	SKCRP	----------	17
LbrM.20.5520	20	SKCRP	----------	15
LbrM.20.5530	20	SKCRP	----------	15
LbrM.21.0160	21	RNI-like, 2xCBSW, CysPc, 3xCBSW	*Low similarity*	178
LbrM.25.1350	25	SKCRP, CysPc	A**EKAYAK**AF**GSY**	80
LbrM.27.0600	27	CysPc, CBSW, CysPc, 2xRPT1, CysPc, CBSW	*Low similarity*	561
LbrM.27.0610	27	CysPc, CBSW, CysPc, RPT1, RPT2, CysPc, CBSW	*Low similarity*	701
LbrM.27.0620	27	CysPc, CBSW	*Low similarity*	60
LbrM.27.2140	27	CBSW, CysPc, 2xRPT1	LEKAYAKFYTLY	623
LbrM.28.2100	28	fragmented CysPC	----------	19
LbrM.30.1980	30	RNI-like, CysPc, CBSW	LEKAYAKSL-GSY	149
LbrM.31.0510	31	SKCRP, CysPc	LEKAYAKLHGSY	98
LbrM.31.0520	31	SKCRP, CysPc	*Low similarity*	162
LbrM.31.0580	31	SKCRP, CysPc	LEKACAKVLGSY	97
LbrM.31.0590	31	SKCRP, CysPc	*Low similarity*	102
LbrM.31.0600	31	CysPc	L*Q*KAYAKVHGSY	53
LbrM.31.0620	31	SKCRP, CysPc	LEKAYAKIHGSY	81
LbrM.32.1060	32	CysPc	LEKAYAKFVGGY	200
LbrM.33.2290	33	MIT, CysPc, CBSW	LEKMLAKLHGGY	123
LbrM.35.0900	35	CysPc	*Low similarity*	122

The ID sequences of the calpain orthologues were retrieved from *L. braziliensis* genome (Genebank ID 718). The identity and similarity of the conserved immunogenic consensus sequence in the cysteine peptidase core (CysPc) domain are presented: bold letters indicate conserved amino acid, residues with strongly similar properties are in italics and underlined and residues with weakly similar properties are in grey. Calpain sequences that contained the CysPc domain but an overall identity lower than 75% with the consensus peptide were designed as ‘low similarity’, and ‘----------’ indicates the absence of the sequence. Genes ID in bold indicate the selection for differential gene expression analyses. ARM: armadillo/beta-catenin-like repeats; CBSW: calpain-type beta-sandwich domain; KISC: kinesin domain; MIT: microtubule interacting and transport domain; RNI-like: regular structure comprising similar repeats; RPT: repeated domain found in de-ubiquitinating proteins; SKCRP: small kinetoplastid calpain-related proteins. 1×, 2× and 3× indicate the number of times that a domain appears in the sequence. Fragmented CysPC stands for short amino acid sequences from the catalytic domain.

Among the 34 predicted calpain-related genes identified in *L. braziliensis* genome, only 23 have the classical CysPc domain (Table). Short amino acid sequences, which correspond to a fragmented proteolytic domain, are present in two putative gene sequences assigned as calpains in the database (LbrM.20.5390 and LbrM.28.2100). Another common and exclusive domain among trypanosomatids is the SKCRPs,^7^ which was identified in 18 predicted genes and stands as the unique domain in eight predicted genes. This domain was found in a diacylated membrane protein, described as small myristoylated protein-1 (SMP-1), which was extensively characterised in *L. major* presenting a specific flagellar localisation.^19^

Besides the calpain CysPc domain found in almost all eukaryotes and few bacteria, calpain-type beta-sandwich domain (CBSW, formerly called ‘C2-domain-like-C2L‘) and penta-EF-hand (PEF) domains are intrinsically associated with classical calpains.^4^ Trypanosomatid calpains, similar to any other single-celled eukaryotes and bacteria, lack the PEF domain, but may contain the CBSW.^7^ Eight predicted genes were found with this domain, out of which only two comprised the core consensus peptide of the CysPc domain, and one of them (LbrM.17.1220) has a kinesin domain (KISC) and three armadillo/beta-catenin-like repeats (ARM) before the CBSW. An orthologous of LbrM.17.1220, which is an orphan kinesin (KIN-E) in *T. brucei*, is reported to play a regulatory role in trypanosome morphology transitions. The kinesin enrichment at the flagellar tip depends on the CBSW domain (also known as m-calpain domain III-like), and the KIN-E depletion in the trypomastigote form causes major morphology changes generating epimastigote-like cells.^20^ Accordingly, the depletion of other *T. brucei* calpain-like gene, such as the repeat-rich CalpGM6, impaired the cellular division and also induced epimastigote-like forms.^21^ Noteworthily, the repeated domains, RPT1 and RPT2, were exclusively found in chromosome 27, which suggests gene duplication events in this chromosome (Table).

**Fig. 1:**
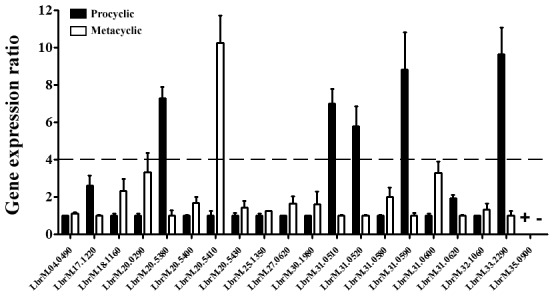
differential gene expression levels of calpain genes between the procyclic and metacyclic stages of *Leishmania braziliensis*. The transcript levels of 20 calpain genes were validated by quantitative reverse transcription polymerase chain reaction (qRT-PCR), and the resulting ratios between procyclics (black fill) and metacyclics (black line) are presented. Actin and protein 8S were used as endogenous controls. The ΔΔCT value of each gene was calculated pair-to-pair between the procyclic and metacyclic stages, and the one with reduced expression was regarded as the reference. The dashed line indicates mRNA ratios with more than 4-fold difference. The symbols + and - indicate a gene with exclusive expression in one of the forms (+). The graph presents the mean of at least four independent experiments performed in triplicate.

Based on the domain distribution across the eukaryote tree and the similarity of domain components in various genes, four calpain architectures CysPc, CysPc-CBSW, MIT-CysPc-CBSW and large transmembrane (TML)-CysPc-CBSW were proposed to be originated early in the evolutionary history of eukaryotes.^6^ The gene expression analysis was focused on genes that presented a conserved CysPc core. We excluded genes with repetitive regions and those related to SKCRP, and included genes with determined functions in related organisms, for instance, the kinesin-like gene, which presents the CBSW domain.^20^ Moreover, *L. braziliensis* calpain-like predicted gene alignment revealed a consensus polypeptide, LEKAYAKLHGSY, in the CysPc domain that shares homology with 13 predicted genes, which prompted us to produce a polyclonal antibody able to recognise all these isoforms [Supplementary data (Fig. 1)].

**Fig. 2:**
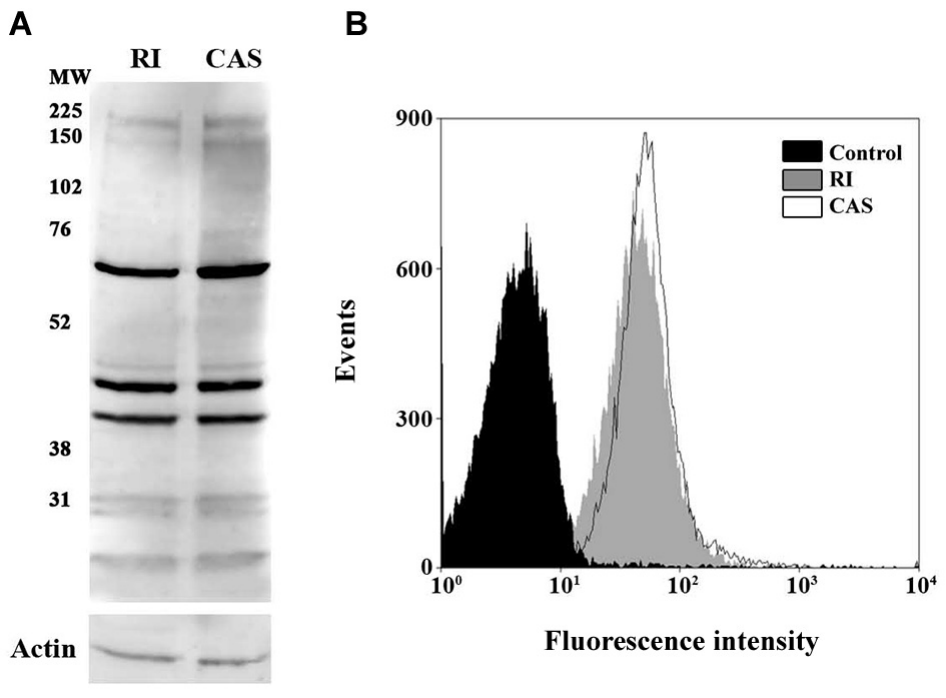
detection of calpain-like molecules in recently isolated or culture-adapted strains of *Leishmania braziliensis* by Western blotting and flow cytometry analyses. (A) Immunoblotting of total cellular extracts of mid-log phase promastigotes of *L. braziliensis* either recently isolated from hamster (RI) or culture-adapted strain (CAS). The membrane was incubated with anti-tritryp-calpain. The relative molecular mass of sodium dodecyl sulfate polyacrylamide gel electrophoresis (SDS-PAGE) protein standards is presented at the left. An anti-actin antibody was used as a control for sample loading in the blots. (B) Flow cytometry analysis of RI parasites (grey fill) or CAS parasites (black line) labelled by anti-tritryp-calpain antibody, autofluorescence control (black fill). Cells treated only with the secondary-Alexa 488 antibody generated similar curves to that observed in the autofluorescence control (data not shown). Representative data of the analysis of 10,000 cells from one out of three experiments are presented. The results are representative of three independent experiments.

*Differential expression of calpain-like genes between procyclic and metacyclicpromastigotes* - Gene expression regulation is an interesting and intriguing phenomenon in trypanosomatids. Constitutive polycistronic transcription of protein-coding genes and *trans*-splicing are known to occur in trypanosomatids. Consequently, trypanosomatid gene expression control mainly occurs at the post-transcriptional level, through untranslated regions (UTR) that impact the mRNA maturation and decay.^22^ Although the analysis of mRNA levels in these organisms sounds controversial, reports reveal a link between the differential expression levels of calpain transcripts and protein expression during trypanosomatid differentiation.^9^ As the biochemical and morphological changes in *Leishmania* from one life cycle to another are presumably the result of programmed changes in gene expression, we compared the mRNA levels of the 19 selected predicted calpain-like genes and the kinesin-like gene (Table) between two *Leishmania* life cycle stages: the proliferative procyclic and the infective non-replicating metacyclic promastigote (Fig. 1). These two life cycle forms were separated by differential centrifugation after attaining the stationary growth phase, as confirmed by flow cytometry [Supplementary data (Fig. 2)]. Primer specificity was confirmed by conventional PCR and sequencing of the amplified product [Supplementary data (Table)], which revealed, for the first time, that the 20 predicted genes are transcribed in *L. braziliensis*.

For the gene expression profile analysis, mRNA ratios equal to or above 4 were considered as differentially expressed in the qPCR assays,^9^ and from the 20 analysed genes, 13 exhibited a constitutive expression profile between the procyclic and metacyclic promastigotes (including the kinesin-like gene), whereas 5 calpain-like genes were highly expressed in procyclics (LbrM.20.5380, LbrM.31.0510, LbrM.31.0520, LbrM.31.0590 and LbrM.33.2290), 1 gene (LbrM.20.5410) was more expressed in metacyclics and only 1 was expressed exclusively in the procyclic forms (LbrM.35.0900; Fig. 1).

Life cycle-specific expression may reveal the calpain orthologue functions in *L. braziliensis*. This study is the first to screen the whole genome of this parasite for calpain identification, and a quantitative PCR was employed to compare the calpain-like gene expression levels between *L. braziliensis* procyclic and metacyclic promastigotes. Previously, an SKCRP expression modulation in *Leishmania* spp. by transcriptomics, proteomics or microarray approaches has been reported.^8^ ^,^ ^10^ ^,^ ^11^ ^,^ ^12^ A microarray analysis of *L. major* promastigotes reported an up-regulation of one calpain-like transcript in procyclic and two in metacyclic stages during the metacyclogenesis;^10^ however, no *Leishmania* genome was available then, and the gene sequence shotgun (GSS) number from the up-regulated sequences of the metacyclic forms presently do not correspond to the calpains, apart from the procyclic up-regulated one (lm73c12), which is an orthologue of LbrM.20.5380, highly expressed in the procyclic stage.

In 2006, Salotra et al.^11^ identified a 2-fold up-regulation of SKCRP (LmjF20.1230) in *L. donovani* parasites isolated from post-kala-azar dermal leishmaniasis patients. This protein corresponds to the aLbrM.20.5450 orthologue. In another approach, a comparative proteomics screen between the anti-monial-resistant and anti-monial-sensitive *L. donovani* strains revealed that SKCRP14·1 (LmjF14.0850) is down-regulated in the resistant strain, and it modulates susceptibility to anti-monials and miltefosine by interfering with drug-induced programmed cell death (PCD) pathways.^12^ Although two *L. braziliensis* calpains (LbrM.14.0820 and LbrM.14.0830) are reported as orthologous sequences of *L. donovani* SKCRP14·1, they were not included in the gene expression analysis because they lacked the CysPc domain, as well as other SKCRPs.

**Fig. 3:**
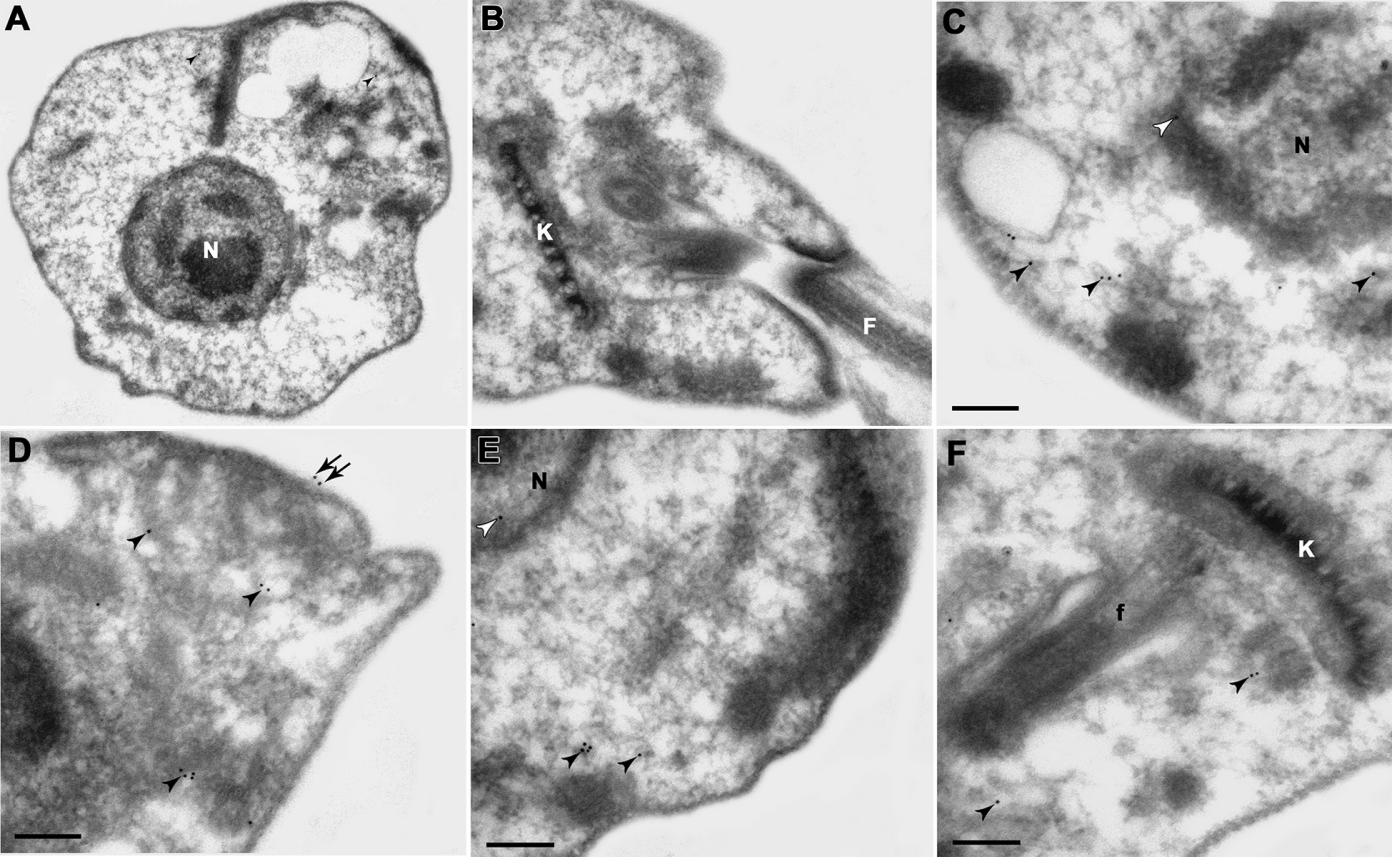
ultra-structural immunocytochemistry of calpains in *Leishmania braziliensis* promastigotes. The labelling was performed in ultra-thin sections incubated with anti-tritryp-calpain antibody, and subsequent incubation with secondary antibody conjugated to a gold particle. (A, B) The omission of the primary antibody indicated the presence of rare unspecific labelling in the cytoplasm (black arrowheads). (C, D, E, F) Promastigotes incubated with anti-tritryp-calpain antibody revealed labelling in the plasma membrane (black arrows), in the whole cytoplasm (black arrowheads), and rare labelling in the nucleus (white arrowheads). The images depicted are representative of three independent experiments. K: kinetoplast; N: nucleus; f: flagellum; Bars = 0.2 μm.

*Protein identification and localisation of promastigote calpain-like molecules* - Aiming to produce a polyclonal antibody to recognise the proteins comprising the conserved CysPC domain to be used as a tool for assessing the global shifts in CysPc-containing proteins in *L. braziliensis*, we selected a consensus polypeptide for peptide synthesis and rabbit immunisation (Table). This antibody was employed in Western blotting analysis, which strongly recognized in *L. braziliensis* mid-log phase promastigote extracts three polypeptides migrating at approximately 70, 45 and 40 kDa. In addition, faint bands were recognised either in the high molecular mass range (~150 and 225 kDa), or low molecular mass range (47 and 31 kDa) (Fig. 2A). This complex pattern was expected since the antibody was raised against a consensus polypeptide found in 13 calpain-like genes with predicted molecular masses ranging from 53 to 623 kDa (Table). Although it is not possible to directly correlate the genes with the reactive polypeptides, the predicted molecular mass is in accordance with the Western blotting data, particularly in the view that certain bands can correspond to a degradation product of the main reactive polypeptides. It is well known that calpains undergo an autolytic conversion in the presence of calcium,^4^ and trypanosomatid calpain orthologues may undergo post-transcriptional changes.^7^ To assay the anti-tritryp-calpain antibody specificity, the antibodies were saturated in a peptide solution (LEKAYAKLHGSY), and were then incubated with the nitrocellulose membranes comprising the transferred *L. braziliensis* proteins, revealing slight reactivity. When the antibodies were pre-incubated with an unrelated polypeptide, no change in the recognition pattern was observed [Supplementary data (Fig. 3)].

Considering that the antibody can only detect global shifts in calpain expression, we considered that it could not improve the quantitative reverse transcription polymerase chain reaction (qRT-PCR) data on the metacyclogenesis process, as the antibody cannot differentiate among the calpain orthologues. Therefore, we compared the overall protein content by western blotting and FACS between RI and CAS parasites, which surprisingly revealed no statistically significant difference (Fig. 2). In previous studies from our group, we reported the recognition of *L. amazonensis* and *T. cruzi* molecules with an antibody raised against *Drosophila melanogaster* calpain-like molecules.^8^ The maintenance of *T. cruzi* in the axenic culture for a long time led to a decreased expression of calpain-like molecules, which suggests a direct relationship between the expression and parasite virulence.^8^ It has been extensively described that peptidases detected by zymography in *L. braziliensis* show decreased expression after long-term cultivation *in vitro*.^23^ Therefore, it is puzzling that the proteins recognised by the anti-tritryp-calpain presented an unaltered expression pattern between the recently isolated parasites from hamster lesions and a culture-adapted strain. Although it does not rule out their involvement in virulence events, this family of proteins might as well be involved in the basic cellular functions, such as cytoskeleton remodelling, a well described function of calpain-like molecules in *T. brucei*.^9^

To determine the cellular localisation of CysPc-containing calpain orthologues in *L. braziliensis* promastigotes, we performed ultra-structural immunolabelling with the anti-tritryp-calpain antibody. Our results revealed the presence of CysPc-containing calpain-like proteins mainly at *L. braziliensis* cytoplasm (Fig. 3C-F), to a lesser extent at the parasite membrane (Fig. 3D), and a faint labelling in the nucleus (Fig. 3C, E). Promastigotes incubated with rabbit pre-immune serum (control) pointed to the presence of rare unspecific labelling in the cytoplasm (Fig. 3A). A similar localisation was reported in *T. cruzi* epimastigotes, where the calpain orthologues were detected mainly in the cytoplasm, with faint labelling in the parasite membrane.^18^ In *T. brucei*, the calpains are distributed in the flagellum and in the cell body, particularly in the cell periphery.^9^ Some of these *T. brucei* orthologues contain N-terminal fatty acid acylation motifs, indicating the association of these proteins with cellular membranes. This N-terminal domain has been also detected in a family of SMPs that are present in *Leishmania* spp., *T. brucei* and *T. cruzi*, being required for the localisation of proteins on the parasite surface or in intracellular membranes, and it is unique to kinetoplastids.^7^ In addition, this is the first study to describe calpain-like molecule association to the nucleus in trypanosomatids. Human calpain 5 has a nuclear localisation, whose functions are yet to be discovered.^24^

**Fig. 4:**
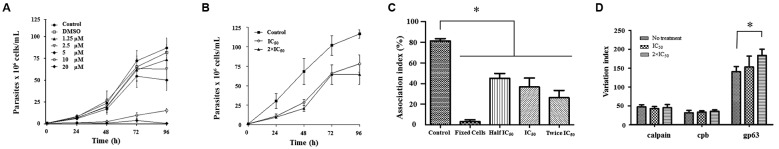
functional studies in *Leishmania braziliensis* by means of the calpain inhibitor MDL28170. (A) The growth pattern of RI promastigotes was followed at 26ºC for 96 h in the absence (control) or in the presence of MDL28170 concentrations ranging from 1.25 to 20 µM. Concentrations as low as 10 µM significantly inhibited the parasite growth from 24 h on in relation to the control (p values < 0.05). (B) Parasites grown for 48 h in the absence or presence of the calpain inhibitor (IC_50_ or 2 × IC_50_/48 h) were washed prior to re-suspension in a drug-free fresh medium and the growth pattern was analysed. (C) Effect of the pre-treatment of *L. braziliensis* RI promastigotes with MDL28170 (½ × IC_50_, IC_50_ and 2 × IC_50_/48 h) during interaction with mouse peritoneal macrophages. Fixed cells were included as an additional control. Results are expressed as the association index of three independent experiments performed in triplicate. (D) Effect of MDL28170 calpain inhibitor on the expression pattern of the calpain, cpb and GP63 in *L. braziliensis* via flow cytometry. RI parasites fixed and permeabilised were treated or untreated with MDL28170 in the concentrations of IC_50_ and 2 × IC_50_/48 h. Cells were then incubated with anti-tritryp-calpain, anti-cpb and anti-GP63 antibodies, and analysed via flow cytometry. The data represent the variation index of the mean fluorescence intensity of the experimental systems in comparison to the auto-fluorescence levels. Data presented are the mean of three independent experiments performed in triplicate; the bars indicate the standard deviation of the mean. * indicates statistically significant difference in relation to control. p < 0.05.

*MDL28170 interferes in parasite proliferation with a long-lasting effect on parasite physiology* - The putative effects of the calpain inhibitor MDL28170 were evaluated on the parasite proliferation, in the early stages of macrophage interaction and during expression of calpain-like molecules and two well-known virulence factors, cpb and gp63.^3^ Moreover, also known as calpain inhibitor III or Z-Val-Phe-CHO, MDL28170 is a potent cell-permeable calpain inhibitor that exhibits neuroprotective effects in numerous rodent neurotrauma models, including spinal cord injury, neonatal hypoxia-ischemia and focal cerebral ischemia.^25^ Here, MDL28170 was added to RI *L. braziliensis* promastigote forms in concentrations ranging from 1.25 to 20 µM, and the cellular growth was compared daily to the control without treatment for 96 h. In parallel, DMSO at the same concentration of MDL28170 highest dilution was added to an alternative control and no effect on the parasite growth behaviour was observed. Our results revealed that MDL28170 arrested the growth in a dose-dependent manner (Fig. 4A). The IC_50_ from each day was calculated and the IC_50_/48 h, which was 6.6 ± 0.4 μM, was chosen for further investigations (Fig. 4A). The anti-leishmanial activity was reversible, since protozoa treated for 48 h with the calpain inhibitor at IC_50_/48h and 2 × IC_50_/48 h (13.2 μM) values resumed growth when cultured in a drug-free fresh medium (Fig. 4B). No significant difference was observed in the growth inhibition between RI and CAS (data not shown). Noteworthily, the parasites treated with MDL28170 at both concentrations presented a longer lag phase than the control cells, lasting up to 48 h (Fig. 4B). The growth rate was comparable to the control cells only from 48 to 72 h, whereas cells treated with 2 × IC_50_/48 h entered prematurely in a stationary phase after 72 h (Fig. 4B). These data clearly indicate that MDL28170 affects the cellular growth and presents a long-lasting effect on the parasite proliferation.

After evidencing the effects of the calpain inhibitor MDL28170 on the promastigote growth pattern, we then treated the parasites with MDL28170 for 1 h prior to the *in vitro* interaction with mouse peritoneal macrophages. RI promastigotes were used in this set of experiments due to the reduced infectivity of CAS parasites (data not shown). Under this experimental condition, the parasites maintained their viability, as judged by their morphology and motility, in which > 95% of the parasites were viable (data not shown). MDL28170 significantly reduced the *Leishmania* intake by macrophages in a dose-dependent manner. DMSO at a dose equivalent to the highest concentration used to dissolve the drug did not promote any significant effect (Fig. 4C), whereas fixed parasites were quickly internalised and destroyed by the macrophages (Fig. 4C). These data indicate that MDL28170 interfered in the early stages of mammalian macrophage infection by *L. braziliensis*. It has been previously reported that MDL28170 can also reduce the infection rate of macrophages by the pre-treatment of *T. cruzi* bloodstream trypomastigotes with MDL28170.^8^ ^,^ ^18^ Moreover, this inhibitor can substantially reduce the intracellular multiplication of several *Leishmania* spp. inside the macrophages, presenting IC_50_ as low as 2.8 μM for *L. braziliensis*, and a toxicity to macrophages of 111.5 μM.^13^

Eventually, we assayed the effects of MDL28170 on the expression pattern of few peptidases (Fig 4D). No significant effect was observed in the calpain and cpb protein levels when the parasites were treated for 48 h with MDL28170 (Fig. 4D); however, GP63 levels increased significantly in the promastigotes treated with 2 × IC_50_/48 h (Fig. 4D). This molecule is a highly abundant zinc metallopeptidase, mainly glycosylphosphatidylinositol-anchored to the parasite surface, which contributes to a myriad of well-established functions for *Leishmania* in the interaction with the mammalian and insect hosts.^26^ We envisage two possible explanations for GP63-like molecules increased levels: MDL28170 could be directly affecting the calpain orthologue functions, and as a physiological compensation, other parasite peptidases are overexpressed;^18^ ^,^ ^27^ or the calpain orthologue inhibition could be producing other non-specific effects on the treated promastigotes, leading to changes in the gene expression of the parasite. A similar compensatory mechanism was reported in *L. amazonensis*, in which both GP63 and cpb were up-regulated after treatment with aspartic peptidase inhibitors.^28^ Nevertheless, the unaltered expression of calpain orthologues by MDL28170 treatment was unexpected, since the treatment of *T. cruzi* and *Phytomonas serpens* with MDL28170 resulted reduced exposition of these molecules.^8^ ^,^ ^18^ In addition, the sub-expression of the target molecules of a drug has been reported in different trypanosomatids that have developed resistance. For instance, Yong et al.^27^ reported that *T. cruzi* epimastigotes from clone Dm28c that were resistant to Z-(SBz)Cys-Phe-CHN2, an irreversible cysteine peptidase inhibitor, presented significantly lower cysteine peptidase activity than that observed in the parental cells, and this fact was accompanied by the reduced expression of cruzipain molecules, the major epimastigote cysteine peptidase. Therefore, the biological significance of the unaltered expression of calpain orthologues in MDL28170-treated *L. braziliensis* needs to be further explored.

**Fig. 5:**
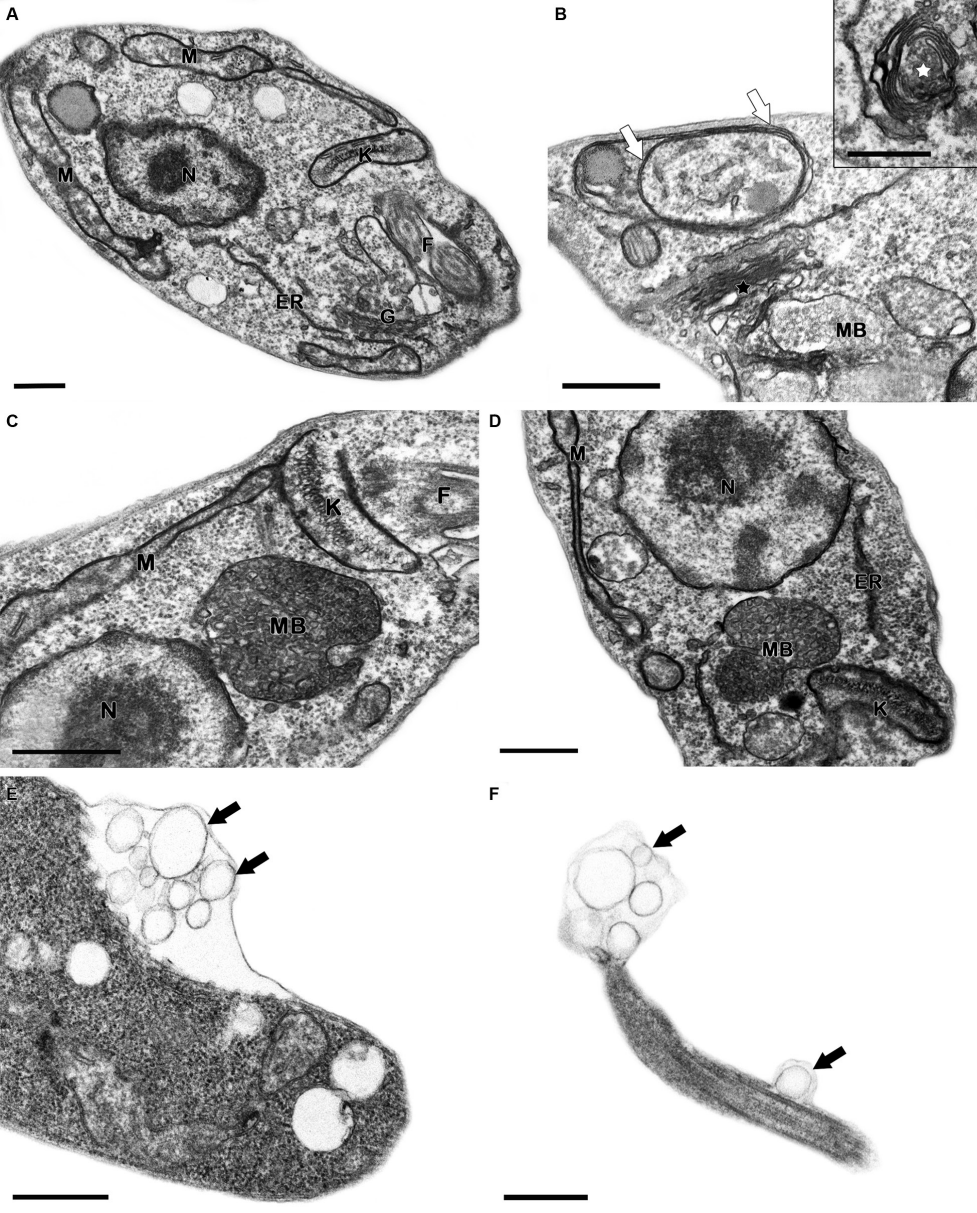
ultra-structural effects of the calpain inhibitor, MDL28170, in *Leishmania braziliensis* promastigotes. (A) Untreated parasite presenting typical morphology. (B-F) Promastigotes treated with 3.5 μM calpain inhibitor for 48 h. (B-D) MDL28170-treated promastigotes revealed concentric membrane structures in the cytosol (white arrows), multi-vesicular bodies (MB), as well as Golgi disruption (black star) and concentric formation of this organelle (white star). (E, F) The treatment with the inhibitor also induced the blebbing of plasma and flagellar membranes (black arrows). ER: endoplasmic reticulum; f: flagellum; G: Golgi; K: kinetoplast; M: mitochondrion; MB: multi-vesicular body; N: nucleus; Bars = 0.5 µm.

*Ultra-structural effects of MDL28170 in L. braziliensis* - Considering that the calpain inhibition by MDL28170 can affect several cellular processes of *L. braziliensis* promastigotes, we analysed the ultra-structure of MDL28170 treated parasites. For this purpose, the morphology of non-treated cells (Fig. 5A) and treated parasites (Fig. 5B-F) was compared. The inhibitor led to the appearance of concentric membrane structures diffused in the cytoplasm (Fig. 5B) and multi-vesicular bodies (Fig. 5B-D). Another organelle affected by the treatment was the Golgi complex, which presented a network disruption in the *trans* region (Fig. 5B). It is possible to observe a concentric distribution of the Golgi cisternae, surrounding portions of cytoplasm (Fig. 5B, inset). The calpain inhibitor also induced a frequent formation of blebbing in the plasma and flagellar membrane (Fig. 5E-F). These effects are indicative of membrane shedding, an usual damage described in trypanosomatids treated with drugs.^29^

In a previous study, our research group demonstrated that *L. amazonensis* promastigotes treated with MDL28170 presented an altered chromatin condensation pattern with apparent loss of nuclear integrity, vacuolisation of the cytoplasm, and disorganisation of the endocytic pathway and a reduced electron density as well as an accumulation of small vesicles.^8^ Here, the ultra-structural alterations observed in treated promastigotes suggest an autophagic process induced by MDL28170 in *L. braziliensis*. Briefly, autophagy comprises a physiological self-degradative pathway that is crucial to maintain the metabolic balance and the recycling of cellular structures during normal cell growth, and its deregulation leads to cell death.^29^ Although poorly understood in trypanosomatids, autophagic phenotype has been described under various stress conditions (drugs, starvation, among others) suggesting that this process is involved in the turnover of damaged structures in the protozoa and is not a cell death pathway.^30^. For instance, when *L. amazonensis* was treated with the aspartic peptidase inhibitors, the ultra-structural alterations compatible with autophagy and membrane shedding were also observed.^28^ Nevertheless, further studies are necessary to better understand the molecular mechanisms of death implicated in MDL28170-induced calpain inhibition in *L. braziliensis*.

*In conclusion* - Over the past few years, the presence of calpain-related proteins in trypanosomatids, responsible for human diseases, has been extensively described. Initially, a classical study employing whole genome analyses reported the presence of a large and diverse family of calpain-related proteins in *T. brucei*, *L. major*, and *T. cruzi*.^7^ Our research group provided the first evidence on the effects of the calpain inhibitor MDL28170 against the aetiological agents of Chagas disease, leishmaniasis, and in classically non-pathogenic trypanosomatids.^8^ ^,^ ^13^ ^,^ ^18^ Here, we identified a wide range of calpain-related domain architectures, and further explored their expression pattern in metacyclogenesis. The functional studies with the calpain inhibitor on *L. braziliensis* added new *in vitro* insights into the study of calpain-related molecule inhibition as an attractive anti-trypanosomatid approach. As up-regulation of several members of the calpain family is involved in a diverse range of biological processes and human diseases, massive efforts have been made to identify selective and potent calpain inhibitors.^5^ Therefore, further studies on trypanosomatid calpain orthologues employing existing drugs developed for the inhibition of human calpains should be carried out, as extreme biochemical selectivity may not be necessary for anti-protozoan drugs due to the inherent biological selectivity in the function and localisation of protozoan peptidases.^5^ Moreover, although more studies are necessary for better understanding the functional role of calpain orthologues in trypanosomatids, our study adds novel data about the genomic context and expression of these molecules, highlighting genes that can be further screened for roles in *L. braziliensis* differentiation process.
