# Supplementary material for: Calpains of Leishmania braziliensis: genome analysis, differential expression, and functional analysis
Source: Mem Inst Oswaldo Cruz. 2019 Sep 23;114:e190147. doi: 10.1590/0074-02760190147 (PMC6759280; doi:10.1590/0074-02760190147)
Supplement: Supplementary file 1 [file 1678-8060-mioc-114-e190147-s.pdf]

TABLE

Gene-specific primers designed for quantitative polymerase chain reaction (qPCR) gene expression analysis

Gene ID	Forward primer	Reverse primer
LbrM.04.0490	TGCAGGTGGAGATGGACGCC	ACTTGGATGCCCCGGAGTCG
LbrM.17.1220	CGGCTACAACGCCTCCGTCT	AGTCCCTCCTGCTCCTCCGA
LbrM.18.1160	GGAGGCAGCTCAACTCCGGT	CATTCCCAGCGCCAACACCC
LbrM.20.0290	TCGTCAGCGAAACGGAGGGG	ACGTACTCTCGCGTGCCAGG
LbrM.20.5380	GACAGGCAGCGACAATGCGG	GCGGCTTGAACGGCTTTCCC
LbrM.20.5400	CAGGATGACAAGCGCGGCAC	GTACTCAGGCGACGGGTGGT
LbrM.20.5410	GCTTTCCCACATCGCGGCTG	GTTGCCGGGGGTACTCAGCA
LbrM.20.5430	GCGATGTGCGGGAGCTTTGG	ATGTCGTAGCGGGAGCACGG
LbrM.25.1350	CCGCAACCATCCGAACGAGC	AAGTCGTAGGCCGGGAAGCC
LbrM.27.0620	GCCTCATGGCGCAAGAACCC	GCATCCGGGTCAGCAAAGCG
LbrM.30.1980	CTGTGCAAGGCAGACGTGCG	AGAAACGCTGCGAGCCCCAA
LbrM.31.0510	AGCGTGCTCTCGGTGCCTAC	GCAAATTCGGGGCCGTCGTG
LbrM.31.0520	GCAAGGTGCTCGGCAAGTGG	TCGGGCGGAAGTTGAGGTCG
LbrM.31.0580	GCAACGCGCTTCTTTGCGCT	CGCCCTTTTTCTGGGTGCGG
LbrM.31.0590	CGCAGGTGATGGTTTGGCCC	GCTGCCTTGGGTCGCACAAC
LbrM.31.0600	TGGGTTACGCTGGAGGCAGG	AGGCCCATCACATACGGGCG
LbrM.31.0620	TATCCGCTGGAGACGCTGCC	TCGCCTCGCGGTTGATCCTC
LbrM.32.1060	ACGGCAGTGGATCAGGGGTG	GCAGAAGCATCGCCTCGCAC
LbrM.33.2290	CGCTACGCTTTTGGTGGGGC	ACGACTCCGGCTTCCGGTTC
LbrM.35.0900	GAGGGGGAGGAAGAGAGGGC	ACCTCAGACGCTGCACGGAG
LbrM.04.1250 (actina)	GGAGGTGCTGTTCAAGCCGT	AGTAGAGCCGCCAGACAGGA
LbrM.24.2160 (S8)	CAGCAGCCACAAGGTCGAGAAG	GGTAGAACTGAAGCTCCGCACC

Actin and protein 8S rod were used as endogenous controls. Each primer was first evaluated for the production of one single product from the template cDNA through conventional PCR followed by gel agarose analysis. Thereafter, the amplified products were purified and sequenced in a Sanger sequencer, as described in Materials and Methods. Eventually, the sequences were aligned against *Leishmania braziliensis* genome to assure that each primer was targeting the gene of interest.

CC BY

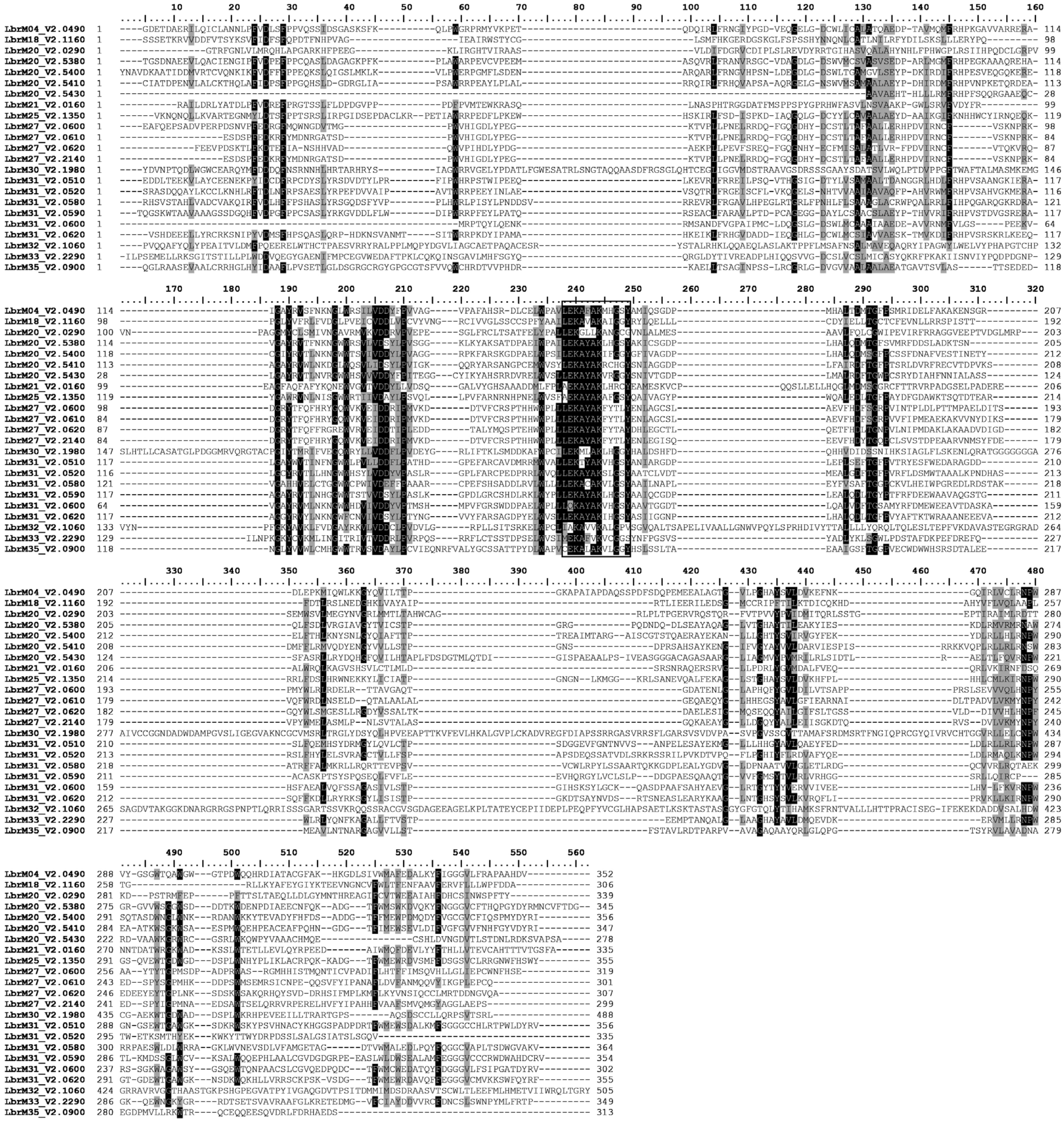

Fig. 1: multiple sequence alignment of the cysteine peptidase core (CysPc) domain of *Leishmania braziliensis* calpain-like proteins. The sequence alignment was performed using ClustalW. TritrypDB ID of the *L. braziliensis* calpain orthologues are at the left. Identical and similar residues are represented in black and grey, respectively. The conserved immunogenic consensus sequence (LEKAYAKLHGSY) is surrounded by a square.

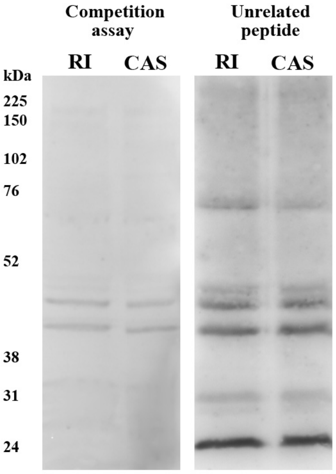

Fig. 2: flow cytometry analysis of *Leishmania braziliensis* population separation by gradient centrifugation. Parasites (1×10^9 cells) were separated in a Ficoll gradient by centrifugation to obtain the metacyclic stages in the supernatant (black line) and the procyclic stages in the *pellet* (grey line). The forward scatter was acquired on a flow cytometer. Representative data analysis of 10,000 cells from one out of three experiments is presented.

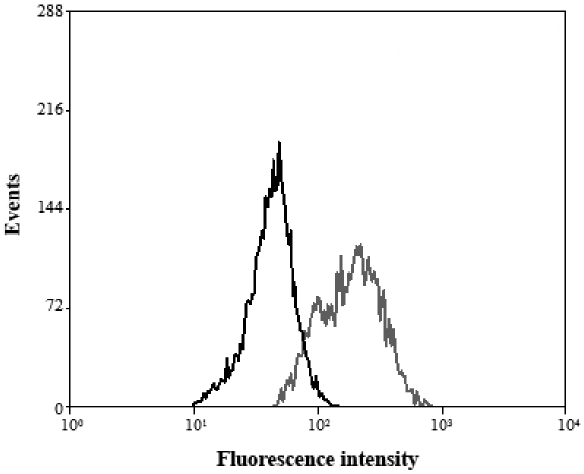

Fig. 3: specificity of the anti-tritryp-calpain against the conserved consensus region of the cysteine peptidase core (CysPc) classical calpain domain. To demonstrate the specificity of the antibody against the consensus region, a 1:500 dilution of anti-tritryp-calpain was incubated in an enzyme-linked immunosorbent assay (ELISA) plate covered with either the consensus polypeptide (LEKAYAKLHGSY) or an unrelated polypeptide (FGFVEEGAEERKAVAELKK). Thereafter, the supernatant obtained after these reactions was collected and incubated in a blotting membrane with cellular extracts of *Leishmania braziliensis* either recently isolated (RI) or culture-adapted strain (CAS). The membranes were incubated with a 1:1500 dilution of the secondary antibody and were subjected to chemiluminescence immunodetection. The relative molecular mass of sodium dodecyl sulfate polyacrylamide gel electrophoresis (SDS-PAGE) protein standards (in kDa) are presented at the left. Alternatively, the absorbance after the standard ELISA protocol was measured, revealing a high capture of the antibody exclusively by the consensus peptide (data not shown).
